# Livedoid vasculopathy in protein S deficiency

**DOI:** 10.11604/pamj.2025.50.77.46735

**Published:** 2025-03-18

**Authors:** Hemant Juneja, Gaurang Aurangabadkar

**Affiliations:** 1 Department of General Medicine, Datta Meghe Medical College, Nagpur, Datta Meghe Institute of Higher Education and Research (DMIHER), (Deemed University), Sawangi (Meghe), Wardha, Maharashtra, India,; 2 Department of Respiratory Medicine, Datta Meghe Medical College, Nagpur, Datta Meghe Institute of Higher Education and Research (DMIHER), (Deemed University), Sawangi (Meghe), Wardha, Maharashtra, India

**Keywords:** Livedoid ulcers, vasculopathy, protein S deficiency

## Image in medicine

A 38-year-old female patient presented to the General Medicine Outpatient Department (OPD) with chief complaints of multiple ulcerations associated with pain over the bilateral lower limbs which was present for 1 month. The patient had initially taken only symptomatic treatment with a topical steroid preparation prescribed by a general practitioner but had no relief. After she presented to us, a rheumatologist's opinion was obtained as we were suspecting vasculitis. All routine investigations were done along with additional tests of protein C, protein S, and factor V Leiden mutation. The protein S levels of the patient were found to be low. Skin biopsy was taken from the edge of the ulcer which was suggestive of vasculopathy with hyalinized dermal vessels. The patient was started on symptomatic therapy with analgesics, compressive bandages, and disease-modifying anti-rheumatic drugs (DMARD). Livedoid vasculopathy is a rare condition that presents with bilateral ulcerative lower limb lesions. It is a multi-faceted disorder that is associated with hypercoagulable states, endothelial damage, and reduced fibrinolysis. It is more commonly seen in female patients. Through this image, we aim to highlight a rare autoimmune differential diagnosis of lower limb ulcers, that may be frequently misdiagnosed as infective lesions.

**Figure 1:**
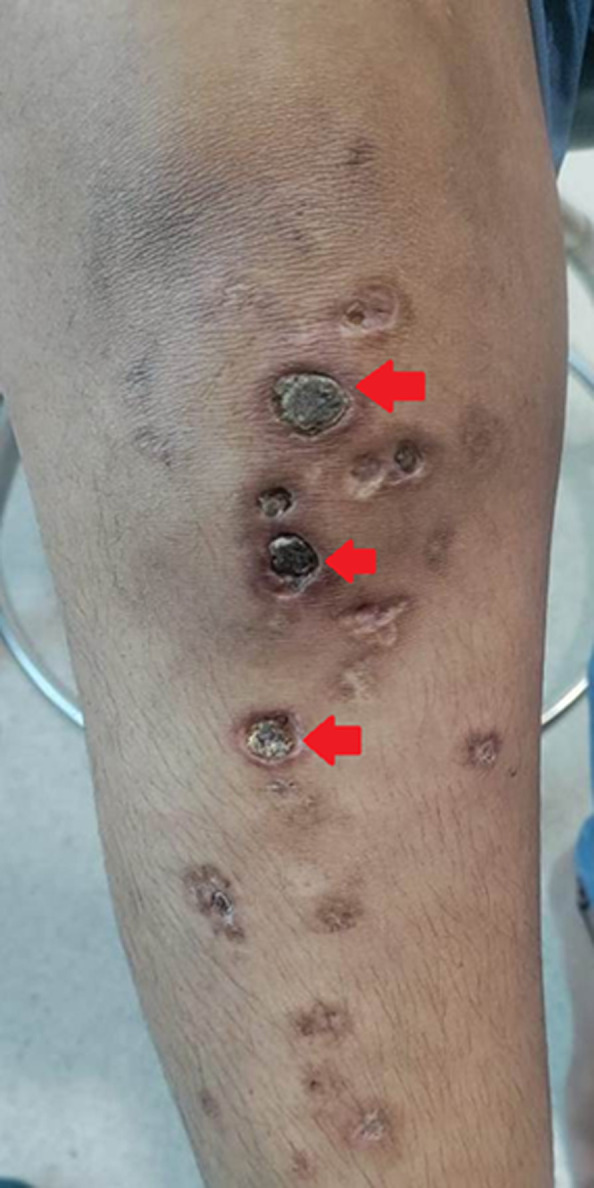
anterior aspect of the right shin showing multiple ulcers with porcelain white scars (red arrows) over the knee and tibial region suggestive of livedoid vasculopathy

